# Expression System Based on an MTIIa Promoter to Produce hPSA in Mammalian Cell Cultures

**DOI:** 10.3389/fmicb.2016.01280

**Published:** 2016-08-17

**Authors:** Anderson K. Santos, Ricardo C. Parreira, Rodrigo R. Resende

**Affiliations:** ^1^Laboratório de Sinalização Celular e Nanobiotecnologia, Departamento de Bioquímica e Imunologia, Universidade Federal de Minas GeraisBelo Horizonte, Brazil; ^2^Instituto NanocellDivinópolis, Brazil

**Keywords:** MTIIa promoter, heavy metals, human prostatic-specific antigen, plasmid vector, recombinant proteins production

## Abstract

Because of the limitations of standard culture techniques, the development of new recombinant protein expression systems with biotechnological potential is a key challenge. Ideally, such systems should be able to effectively and accurately synthesize a protein of interest with intrinsic metabolic capacity. Here, we describe such a system that was designed based on a plasmid vector containing promoter elements derived from the metallothionein MTIIa promoter, as well as processing and purification elements. This promoter can be induced by heavy metals in a culture medium to induce the synthesis of human prostate-specific antigen (hPSA), which has been modified to insert elements for purification, proteolysis, and secretion. We optimized hPSA production in this system by comparing the effects and contributions of ZnCl_2_, CdCl_2_, and CuSO_4_ in HEK293FT, HeLa, BHK-21, and CHO-K1 cells. We also compared the effectiveness of three different transfection agents: multi-walled carbon nanotubes, Lipofectamine 2000, and X-tremeGENE HP Reagent. hPSA production was confirmed via the detection of enhanced green fluorescent protein fluorescence, and cell viability was determined. The expression of hPSA was compared with that of the native protein produced by LNCaP cells, using enzyme-linked immunosorbent assay and sodium dodecyl sulfate polyacrylamide gel electrophoresis. X-tremeGENE reagent, the BHK-21 cell line, and CuSO_4_ showed the highest hPSA production rates. Furthermore, BHK-21 cells were more resistant to the oxidative stress caused by 100 μM CuSO_4_. These results suggest that the proposed optimized inducible expression system can effectively produce recombinant proteins with desired characteristics for a wide range of applications in molecular biology.

## Introduction

The production of highly complex proteins is preferably performed in target expression eukaryotic cell cultures. Insect cells transformed by baculovirus infection have gained popularity, owing to their high growth rate and the scale on which they can be produced. These cells are particularly useful because of their resistance to metabolic stress. However, other limitations related to baculovirus infection mechanisms and the incorrect folding and secretion of proteins limit their use for certain types of proteins (Treanor, 2009; Walsh, 2014).

Metallothioneins were discovered in 1957 as cadmium protein ligands in equine kidneys. They are small, cysteine-rich proteins that play a fundamental role in the homeostasis of heavy metals. Transcription is regulated by these metals, but other stimuli such as hypoxia, oxidative stress, or hormones have been described (Kagi and Valee, 1960; Haslinger and Karin, 1985).

The promoter region of most metallothionein genes comprises short sequences of DNA collectively known as MREs, AREs, or GREs (Gunther et al., 2012). In particular, MREs are highly conserved between invertebrates and vertebrates (Yan and Chan, 2004). MREs are highly expressed in multiple combinations. Usually, these elements are found upstream of heavy metal response promoters. They were originally found in metallothioneins (Dalton et al., 1994).

The sequence TGCRCNC (R = A or G and N = any nucleotide) is highly conserved and is considered an MRE. From this, the existence of specific transcription factors that regulate the response to heavy metals was suggested. This was confirmed by Séguin and Prévost (1988), who discovered that MTF-1 was a transcription factor with a high-affinity zinc finger DNA domain.

The formation of ROS and the increased expression of proteins responsible for stabilization results in the recruitment of cofactors and auxiliary transcription factors involved in MTF-1 expression (Li et al., 2008). It is also believed that transition metals interact directly with a metalloregulatory protein (called a “zinc” sensor), possibly MTF-1 itself, and that this interaction leads to a conformational change of the protein, thereby altering its affinity for RNA/DNA and promoting gene activation (Smirnova et al., 2000).

Some studies investigated the MTF-1 activation capabilities that are directly dependent on the number of MRE copies in the promoter region of MTI and MTIIa. It was discovered that MTF-1 can be activated independently of MRE (Suzuki and Koizumi, 2000).

The presence of only one MRE is sufficient to support and recruit other transcription factors required for the elevated production of a transcript (Suzuki and Koizumi, 2000). Based on this work, it is assumed that, owing to the high affinity of MTF-1 for MREs, a small heavy metal stimulus can initiate high-performance transcription. It is believed that the minimum presence of heavy metals in an *in vivo* cellular environment requires a rapid and sharp response for resolution, which explains the rapid and robust response provided by MTF-1.

An induced expression model with elements that facilitate the recovery of the recombinant protein and a method to monitor the whole expression process of a fluorescent reporter in a simplified form can facilitate the production of complex proteins with a large number of post-translational modifications and complex folding. These would increase the chance of maximum function and recognition of these proteins by the original organism. Thus, they garnered scientific and technological interest.

Inducing protein expression using heavy metal is innovative. In this study, the MTIIa promoter reduced was associated with the gene of interest and a fluorescent reporter gene, purification elements (e.g., His-tag), an extracellular secretion signal peptide, and a proteolytic cleavage site for TEV protease. This method allowed us to generate a set of factors that guaranteed a high rate of production and recovery of the protein in the culture medium, especially for proteins with high molecular complexity such as hPSA used as a model in this study.

## Materials and Methods

**FIGURE 1:**
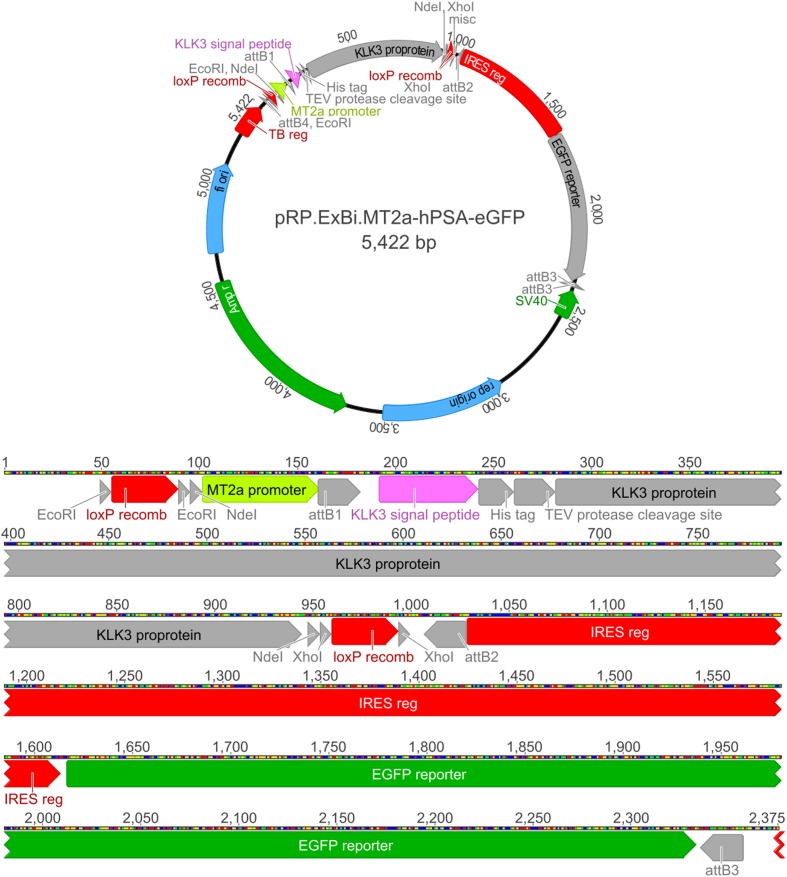
**pRP.ExBi-MTIIa-hPSA-eGFP vector *Eco*RI, *Nde*I, and *Xho*I restriction sites correspond to endonucleases with the same names.** Recomb LoxP recombination are LoxP sites for Cre-LoxP recombination. F1 ori represents the bacterial origin of replication. The TEV protease cleavage site is a proteolytic cleavage site for TEV protease. Amp R is the β-lactamase gene, which is used for the selection of recombinant bacteria. It corresponds to the SV40-derived eukaryotic origin of replication of simian virus 40. The eGFP reporter corresponds to the fluorescent eGFP reporter gene. The reduced promoter MTIIa (or MTIIa) is followed by the hPSA sequence, which includes the signal peptide, the His-tag and the cleavage site for TEV protease (TEV protease cleavage site) in its initial region. After the target gene, an IRES region and the eGFP reporter gene were included. Recombination Cre/LoxP sites were included flanking promMTIIa-hPSA.

We used the hPSA sequence (1906 bp; NM_001030047.1) as the target gene and pRP.ExBi (Cyagen Biosciences Inc., Sunnyvale, CA, USA) as the vector. The reduced (371 to 60 bp) MTIIa promoter region (X00504.1) was inserted before the target gene. The pRP.ExBi backbone allowed the insertion of the promoter sequence of the gene of interest and a reporter gene. The MTIIa promoter (60 bp) has multiple copies of *cis*-regulatory elements and is involved in the MRE for MTF-1 binding (Jervis and Kilburn, 1996). Besides possessing the target gene sequence, the insert contains hPSA purification and secretion elements such as a His-tag, a cleavage site for TEV protease, and an extracellular secretion sequence. The reporter gene, eGFP, was then inserted (700 bp). The obtained plasmid was synthesized by Cyagen Biosciences Inc., and is referred to as pRP.ExBi-MTIIa-hPSA-eGFP (**Figure 1**. Plasmid complete sequence can be seen on **Supplementary Data Sheet S1**).

pRP.ExBi-MTIIa-hPSA-eGFP was cloned in *Escherichia coli* Stbl3 and selected by ampicillin resistance (100 μg/mL) in SOC medium. The strain was grown under orbital agitation (200 rpm) at 37°C for 16 h in 10 mL of SOC culture medium. Immediately after culture, it was centrifuged at 6800 × *g* for 2 min, and the obtained pellet was purified using a GeneJET Plasmid Miniprep Kit (Thermo Fischer Scientific, Waltham, MA, USA) following the manufacturer’s instructions.

### Maintenance and Mammalian Cell Culture

The HEK293FT (ATCC CRL-1573), HeLa (ATCC CCL-2), LNCaP (ATCC CRL-1740), CHO-K1 (ATCC CCL-61), and BHK-21 (ATCC CCL-10) cell lines were maintained in culture in DMEM supplemented with 10% FBS, 2.5 U/mL penicillin, 2.5 μg/mL streptomycin, and 5 μg/mL gentamicin (Thermo Fischer Scientific), and incubated at 37°C in a humidified atmosphere containing 5% CO_2_.

### Transfection of Cell Lines with pRP.ExBi-MTIIa-hPSA-eGFP

Three agents were used for transfection: MWCNT, Lipofectamine 2000 (Thermo Fischer Scientific), and X-tremeGENE HP Reagent (Roche, Basel, Switzerland). All experiments were performed in triplicate. In a microfuge tube, 100 μL of MWCNT 0.25 mg/mL was added to 300–500 ng of plasmid DNA (pDNA) and kept in an ultrasonic bath at 40 kHz and 120 W for 30 min at 4°C, then kept for 10 min at 4°C. The MWCNT-pDNA complex was then added to the well containing the cells to be transfected into a final volume of 2 mL, homogenized by rotational movement, and incubated at 37°C in a humidified atmosphere containing 5% CO_2_.

The X-tremeGENE HP Reagent (Roche) was used to transfect the cell lines according to the manufacturer’s specifications with modifications like those used for Lipofectamine 2000 (Thermo Fischer Scientific). Six microlitres of each reagent were incubated with 1 μg of pDNA in 100 μL of DMEM supplemented with 1 mM sodium pyruvate and 0.1 mM non-essential amino acids for 10 min and added to the cells. Twenty-four hours before the transfection procedure, 1 × 10^4^ cells/well were seeded in a 6-well plate corresponding to 70% confluency at the beginning of the transfection. DMEM supplemented with 1 mM sodium pyruvate and 0.1 mM non-essential amino acids was used for culture.

### Cell Viability

Cell viability was determined by using an alamarBlue^®^ Cell Viability Assay (Thermo Fischer Scientific) according to the manufacturer’s instructions in duplicate. Briefly, 1 × 10^4^ transfected cells were incubated with 100 μM CuSO_4_ in combination with 20 μL of the alamarBlue^®^ reagent for 4 h at 37°C in a humidified atmosphere containing 5% CO_2_. Metabolically active cells reduce the dye to its fluorescent form, and the fluorescence was measured using a Varioskan Flash Multimode Reader (Thermo Fischer Scientific) at 570 nm/600 nm (excitation/emission). Cell viability was determined by setting the maximum (100%) for the cells without heavy metal treatment.

### Induction of Expression Mediated by Heavy Metal Salts

The stock solutions (10 mM) consisted of ZnCl_2_, CuSO_4,_ and CdCl_2_ salts. Each solution was used at concentrations of 1, 10, and 100 μM. In the case of CuSO_4_, the solution was also used at 500 μM. The solutions were simultaneously added at the transfection stage of the procedure in triplicate.

### Analysis of Protein Expression

The fluorescence intensity generated by eGFP and ZSGreen was measured using a Varioskan Flash Multimode Reader (Thermo Scientific) at 497 and 505 nm for excitation and emission, respectively. The fluorescence images were captured using a Nikon Eclipse fluorescence microscope Ti (Nikon Instruments Inc., Melville, NY, USA) and an EVOS^®^ FL Imaging System (Thermo Fischer Scientific). The ratio between the fluorescent and non-fluorescent cells was quantified.

Verification of hPSA expression was carried out by SDS-PAGE on 12% acrylamide. The samples of supernatants were fractionated and prepared by heating for 5 min at 98°C. The samples were then pipetted into the wells of the gel and electrophoresis was carried out at 100 mV, while the samples passed through the stacking gel. Subsequently, 120 mV was used for sample migration in the gel and protein resolution. After electrophoresis, the gel was stained with Coomassie Brilliant Blue and treated for 15 min with 5% glycerol for subsequent drying. The gel was de-stained and documented.

The enzyme-linked immunosorbent assay was carried out using a Human PSA ELISA Kit (Sigma–Aldrich, St Louis, MO, USA) following the manufacturer’s specifications to quantify hPSA in the total cell culture supernatants. hPSA dilutions (10:24, 25:6, 64, 160, 400, 1000, and 2500 pg/mL) were pipetted into the wells to generate the standard curve. Supernatant samples were also pipetted into the wells. The wells were then washed and the anti-biotinylated hPSA was added.

## Results

Sequentially, protein of interest and gene reporter expression temporally controlled when a heavy metal salt is added to the medium, and then the preproprotein (signal peptide – His tag – TEV site – protein) can be delivered to medium. Thereby, the medium is harvested and through a Ni^2+^-affinity chromatography column to His tag, a fraction of protein is obtained. This fraction is so digested by TEV protease, releasing a peptide (signal peptide-His tag-TEV site) and protein of interest.

### hPSA Protein Expression

**FIGURE 2:**
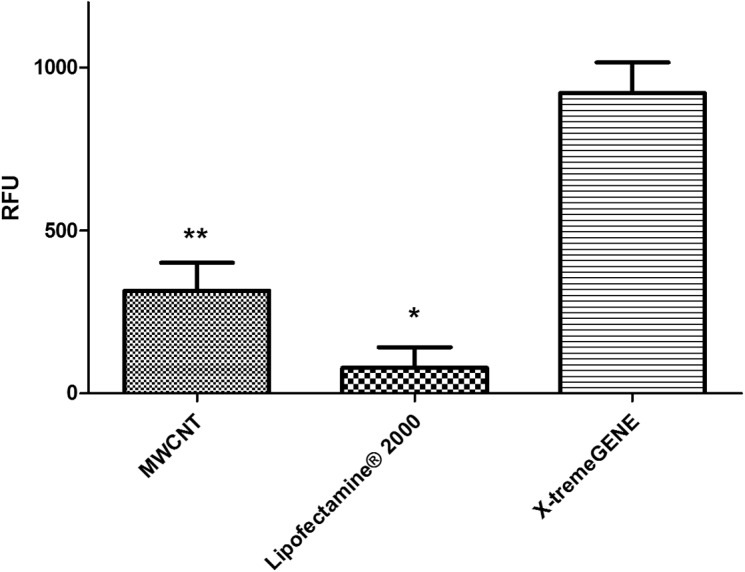
**Analysis of the different transfection methods MWCNT, Lipofectamine 2000, and X-tremeGENE transfection agents were used and the efficiency of protein expression was compared by measuring the fluorescence of pZSGreen-N1 and pRP.ExBi-MTIIa-hPSA-eGFP in HEK293FT cells under the same conditions.** The expression control served to determine the ZSGreen transfection quality of the reference method, which was compared with the expression of eGFP from pRP.ExBi-MTIIa-hPSA-eGFP. Error bars indicate standard deviation ^∗^*p* < 0.05 between Lipofectamine eGFP and ZSGreen. ^∗∗^*p* < 0.05 between MWCNT and X-tremeGENE eGFP [one-way analysis of variance (ANOVA) followed by Tukey’s *post hoc* test].

**FIGURE 3:**
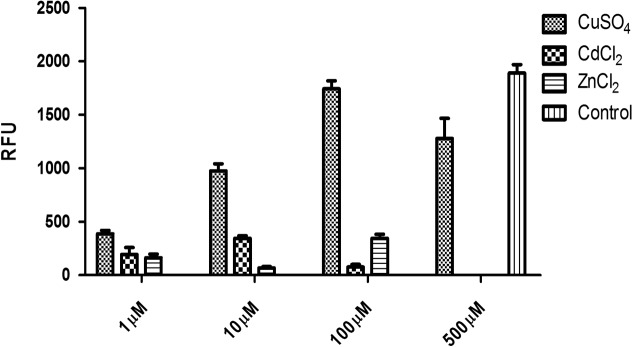
**Heavy metal-induced expression of hPSA in pRP.ExBi-MTIIa-hPSA-eGFP hPSA expression levels in the presence of 1, 10, and 100 μM CuSO_4_, CdCl_2_, and ZnCl_2_, and 500 μM CuSO_4_, 48 h after induction.** Error bars indicate standard deviation.

The eGFP reporter was co-expressed with hPSA and its detection was used as a direct confirmation of the expression of hPSA protein measured by ELISA and SDS-PAGE. Three transfection agents were investigated: MWCNT, liposomal complexes [Lipofectamine (Thermo Scientific)], and non-cationic liposomal complexes (X-tremeGENE (Roche; **Figure 2**). The best transfection system was identified by measuring the fluorescence generated by the eGFP reporter present in the pRP.ExBi-MTIIa-hPSA-eGFP vector in HEK293FT cells. Fluorescence analysis of ZSGreen expressed by the pZSGreenN1 vector was used as a positive control (under the same conditions). Fluorescence was only considered satisfactory when using non-cationic liposomal complexes. The results obtained with X-tremeGENE (Roche) were the best among the transfection agents tested and therefore the following experiments were performed using X-tremeGENE. After transfection, we determined whether the inducer agent would induce high protein expression. CuSO_4_, CdCl_2_, and ZnCl_2_ were evaluated at 1, 10, and 100 μM. CuSO_4_ was also tested at 500 μM. These values were chosen because they were in the non-toxic concentration range in cell culture. The fluorescence increased in cells treated with CuSO_4_, reaching a maximum after 100 μM heavy metal treatment (**Figure 3**).

### Expression Over Time

**FIGURE 4:**
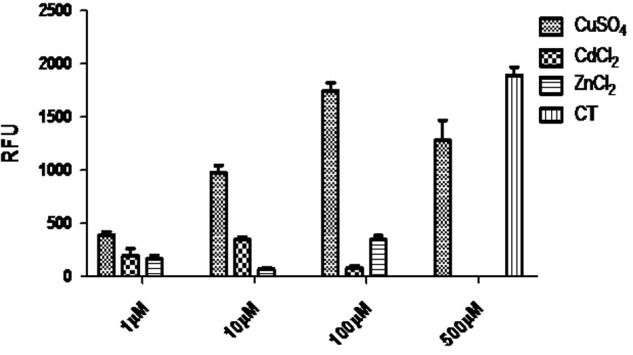
**Effect of induction on the eGFP expression mediated by 100 μM CuSO_4_ in HeLa cells transfected with pRP.ExBi-MTIIa-hPSA-eGFP.** Samples were collected at 0, 24, 36, 48, and 72 h after induction and the fluorescence of eGFP was measured. Non-induced cells were used as controls. Error bars indicate standard deviation.

The production of recombinant hPSA was accompanied by the expression of the eGFP reporter in transfected HeLa cells using the X-tremeGENE method in the presence of 100 μM CuSO_4_. The data were measured at 0, 24, 36, and 72 h after transfection (**Figure 4**). From 48 h, all tested cells presented the maximum fluorescence in the presence of 100 μM of CuSO4. Basal eGFP expression was detected in the no induction control.

### Fluorescence Microscopy

**FIGURE 5:**
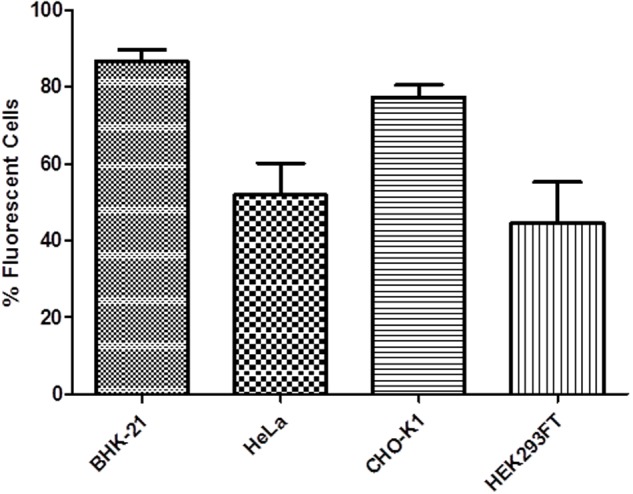
**Percentage of fluorescent cells transfected with pRP.ExBi-MTIIa-hPSA-eGFP.** Error bars indicate standard deviation.

The fluorescence microscopy images showed the expression of the eGFP reporter, both visually and qualitatively. HEK293FT, HeLa, BHK-21, and CHO-K1 cell lines were transfected with pRP.ExBi-MTIIa-hPSA-eGFP, and the pZSGreen-N1 vector was used as a control (**Figure 5**). All cell lines exhibited fluorescence and demonstrated morphologic abnormalities in comparison with untransfected cells.

### SDS-PAGE Analysis

**FIGURE 6:**
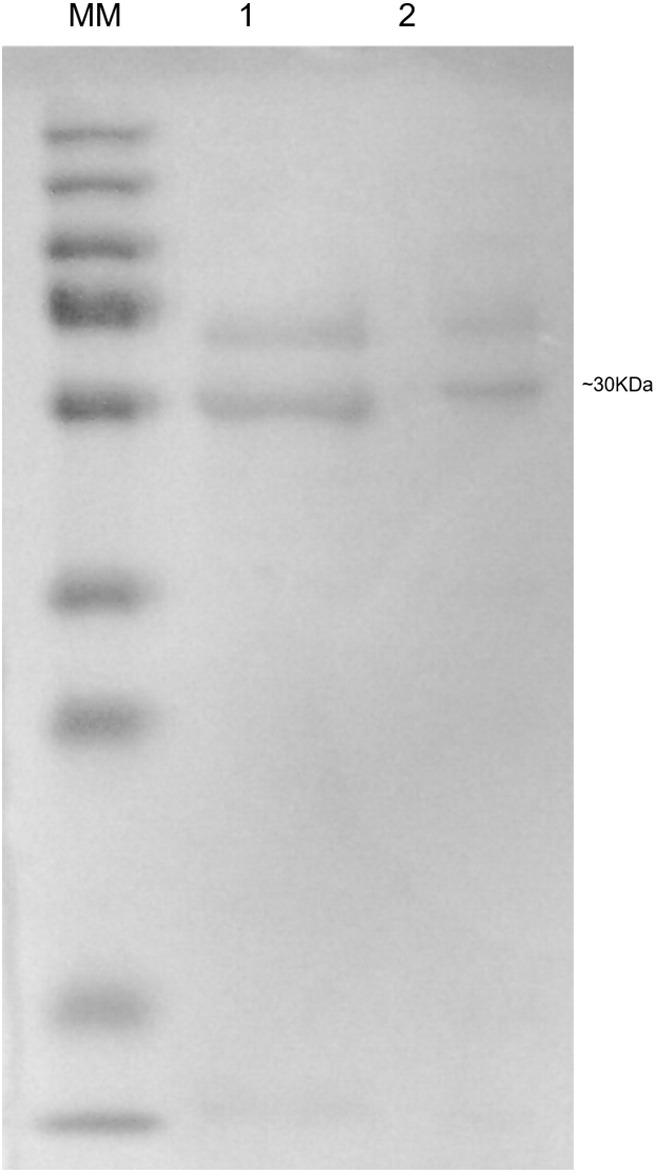
**Sodium dodecyl sulphate polyacrylamide gel electrophoresis (SDS-PAGE) of hPSA.** The MW pit corresponds to the molecular mass standard used, lane 1 corresponds to the protein fraction of the supernatant from LNCaP cell culture, and lane 2 corresponds to the supernatant from the recombinant BHK-21 cell culture.

SDS-PAGE was performed to verify the size of the hPSA recombinant protein produced by BHK-21 cells and the native protein naturally produced by LNCaP cells (**Figure 6** lanes 1 and 2, respectively). Two bands were visible, one at approximately 30 kDa, the size of hPSA, and another of approximately 50 kDa, which could correspond to a protein complex obtained during the fractionation of the supernatants.

### hPSA ELISA

The direct production of hPSA was quantified using ELISA as a primary test. Besides quantifying hPSA expression, this test also provided information about recombinant proteins that maintained their antigenicity and conformational structure.

**FIGURE 7:**
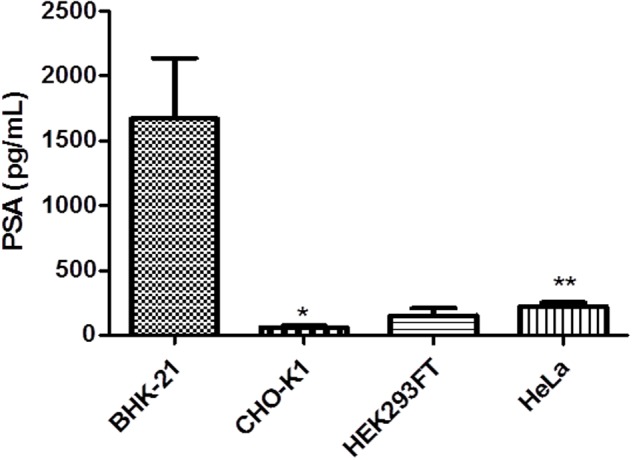
**Enzyme-linked immunosorbent assay (ELISA) of recombinant hPSA from BHK-21, HEK293FT, HeLa, and CHO-K1 cells.** Error bars indicate standard deviation. At 72 h after transfection, cells were induced with 100 μM CuSO_4_ to measure the concentration of hPSA. ^∗^*P* < 0.05 between CHO-K1 and HEK293FT cells, ^∗∗^*P* < 0.01 between HEK293FT and HeLa cells (ANOVA followed by Tukey’s *post hoc* test).

The assay was performed 72 h after transfection of BHK-21, HEK293FT, HeLa, and CHO-K1 cells induced with 100 μM CuSO_4_ and in the un-induced controls. Levels of hPSA in un-induced controls were subtracted from those in induced cells (**Figure 7**).

The BHK-21 cell line demonstrated greater hPSA production capacity in inducing conditions, approaching 1600 pg/mL. As a cumulative effect, the maximum expression achieved was 50 ng/mL hPSA with 1 × 10^4^ cells/well.

### Cell Viability

Induction of hPSA expression by heavy metals could induce cellular death due to oxidative stress generated in the presence of heavy metals. Therefore, cell viability should be assessed during exposure to heavy metals.

**FIGURE 8:**
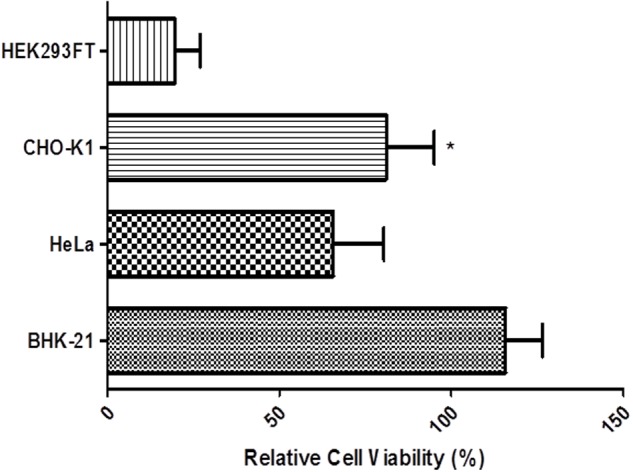
**Cell viability measured with the alamarBlue^®^ assay in BHK-21, HEK293FT, HeLa, and CHO-K1 cells.** At 72 h after transfection, cells were induced with 100 μM CuSO_4_ and the viability was measured. Error bars indicate standard deviation. ^∗^*P* < 0.05 between CHO-K1 and HeLa cells (ANOVA followed by Tukey’s *post hoc* test).

The cell viability assay using the alamarBlue^®^ (Thermo Scientific) method allowed us to identify the strain that was most resistant to heavy metal treatment. The experiment was carried out 72 h after transfection in four cell lines: BHK-21, HEK293FT, HeLa, and CHO-K1 induced by 100 μM CuSO_4_ (**Figure 8**). Cell viability remained constant in CHO-K1 and BHK-21 cell cultures throughout the experimental period. In particular, in BHK-21 cells, the survival rate was greater than 100% compared with the untreated culture. BHK-21 cell line features a great adaptive ability and this may explain why the survival range was superior to that of controls.

## Discussion

The purposed vector was designed to be adaptable to all mammalian genes which code for high level of posttranscriptional modification proteins. To our knowledge this is the first time that reduced MTIIa promotor is used together with sequences that facilitate secretion and purification from the culture medium.

Expression systems for therapeutic recombinant proteins should allow the production of proteins that retain both the physicochemical properties and the conformational structure of the native protein. Meanwhile, the efficiency of expression must be guaranteed for as long as possible by the transfected cells. This balance requires modifications of the target gene to include sequences that allow the separation of the protein in the culture medium.

The presence of LoxP sites was programmed to be easily replaced by other gene of interest, this guarantee that a same vector can be useful to several applications.

During oxidative or hormonal stimulus, or in the presence of heavy metals, metallothionein promoters, specifically MTIIa and MTI, are described in the literature as highly active genomic regions (Gunther et al., 2012). The size of the promoter sequence is ≥5 kb with repeat motifs of 4–8 MREs distributed near the TATA box (Haslinger and Karin, 1985). The activation of gene expression by transcription factors with high affinity for MREs is not well defined. The main element in this process, MTF-1, appears to play a significant role when at least moderate concentrations of heavy metals are present in the cellular environment (Smirnova et al., 2000; Shang et al., 2002).

These promoters present features that demonstrate high efficiency for inducing expression, which is stimulated by the MTIIa promoter, suggesting that their use for the production of recombinant proteins is a plausible and highly profitable strategy (Kushner et al., 1986).

Particularly in industry, the use of mammalian cell cultures for the production of recombinant proteins requires a very large volume of growth medium containing an initial quantity of cells, which do not necessarily express high yields of proteins. Recombinant cultures using constitutive promoters have a tendency for gene silencing and higher metabolic stress compared with cultures using inducible promoters (Takekoshi et al., 1993; Barrow et al., 2006; Johnen et al., 2012). Based on the current view, protein expression would be induced in stages where the culture is already established and more resistant to adverse conditions that may arise during the growth process itself or at induction. Expression mediated by MRE/MTF-1 presents additional advantages when compared with systems based on constitutive or inducible promoters, particularly from the industrial point of view. Chief among those advantages is their capacity for activation at low heavy metal concentrations. For MREs, handling involves simple molecular biology tools that use a promoter base. Finally, the low cost of the inductors is an attractive factor for their use compared with hormones and drugs, which are most commonly used (Jervis and Kilburn, 1996; Liao and Sunstrom, 2006; Santos et al., 2007).

Some studies attempted to analyze, improve, and even reduce the MTIIa promoter sequence to the fundamental elements that retain its characteristics. Handling focuses primarily on the number of MREs in the sequence (Park et al., 2000; Yan and Chan, 2004; Santos et al., 2007).

Suzuki and Koizumi (2000) used this principle to propose the use of the MTIIa promoter, and found that its sequence is capable of recruiting MTF-1 independently and, thus, maintaining a constant rate of expression of the target gene. Based on this work, we propose the use of a promoter-terminal region corresponding to an MRE at the transcription start site, part of the region that was validated by the aforementioned group. The model uses 1–4 copies of the MRE of the MTIIa promoter. The induction model in this study, in addition to its validation in different cell lines, proposes an alternative, more efficient method for obtaining the protein of interest, including purification elements that enable scale-up to a bioreactor to produce the tumor marker, hPSA.

The gene encoding hPSA was modified for high efficiency expression by adding sequences for the His-tag and a TEV protease for proteolytic cleavage, and by relocation of the secretion signal peptide. The hPSA gene naturally possesses a region encoding a secretion signal peptide. This region was relocated upstream of the His-tag_TEVsite_hPSA sequences, thereby ensuring that the secretion signal would be maintained.

The protein processing order prevents new sites with antigenic potential from forming artificially. In the newly transcribed protein, which is called a pre-pro-protein, the N-terminal region presents a secretion signal peptide followed by a His-tag and a TEV cleavage site. The formed protein is intended for export to the extracellular medium and the signal peptide is removed by peptidases involved in cell addressing.

Collection and fractionation of the culture medium allows us to obtain a protein moiety mainly by chromatographic techniques such as size exclusion and ion exchange. At this stage, the pro-protein only has the His-tag and the TEV site, and an affinity purification column with a nickel complex can be used. The His-tagged protein forms a stable complex with the nickel ion that is immobilized in the column resin. Thus, it is possible to obtain a recombinant protein fraction in larger amounts and of higher purity.

However, the His-tag sequence provides a novel immunogenic site that should be removed. The region between the signal peptide and the TEV site forms an antigenic determinant (in accordance with the EMBOSS Protein using the Kolaskar and Tongaonkar (1990). This algorithm is based on the occurrence of amino acid residues in experimentally determined epitopes. Therefore, the recombinant protein fractions must still go through a new proteolytic processing step to obtain a protein without the His-tag that is similar to the native protein.

Tobacco etch virus protease is a highly specific endopeptidase selective to the ENLYFQS that is widely used to fragment fused proteins expressed in both eukaryotes and prokaryotes (van den Berg et al., 2006). In the case of hPSA, a serine residue is the only trace formed after treatment with the TEV protease. Shortly thereafter, the obtained recombinant protein is homologous to the native.

Some additional points were considered in the construction of the expression vector: very large sequences were avoided in the plasmid because they decrease bacterial replication efficiency, resulting in a low copy number. This is also the reason for reducing the size of the MTIIa promoter region. Similarly, a reduction in the promoter would decrease the cost of plasmid synthesis, because large inserts increase costs and affect efficiency.

Human prostate-specific antigen is highly glycosylated; therefore, the decision to express the protein in mammalian cells depends on the fact that any changes in the glycosylation pattern could dramatically alter the affinity of the antibody for the protein because the antibody recognizes glycosylation sites or sites near to them (Shively and Beatty, 1985; Kojima et al., 1993; Zheng et al., 2011; Andersen et al., 2012).

The manipulation of sequences for use as a backbone for future projects explains the inclusion of restriction sites for endonucleases and, particularly, the inclusion of LoxP sites. The requirement for deletion, inversion, and addition of sequences can be readily met via Cre-LoxP recombination or a single digestion with restriction enzymes.

Protein expression was considered efficient and responsive within the 1st hour after transfection and induction. Zinc, cadmium, and copper salts were used based on the current knowledge regarding the MTF-1. The first step was to define the transfection system because the target gene is of a considerable size, which dramatically affects how the exogenous DNA enters the cell.

Lipofectamine and carbon nanotubes have been proven very effective transfection agents (Tonelli et al., 2015). The latter are widely used for transient expression of plasmid vectors (Ladeira et al., 2010; Tonelli et al., 2014). The control vector behaved as expected. The expression of control was effective, but, in the pRP.ExBi-MTIIa-hPSA-eGFP vector, its presence proved ineffective.

Multi-walled carbon nanotubes have the ability to deliver larger DNA fragments by compressing the material to be complexed and decreasing the contact area (Tonelli et al., 2014). Previous studies showed that the MWCNT-DNA complex can move more effectively through the cell membrane, but the synthesized vectors were larger than those used in this methodology were. Parameters such as nanotube size and the area of functionalized carboxylated groups were not adapted to large DNA sequences and, therefore, the expression of pRP.ExBi-MTIIa-hPSA-eGFP was not as pronounced.

Cationic liposomal complexes have the ability to neutralize the negative charges of DNA and facilitate passage through the plasma membrane. Immediately after the penetration of the membrane, the product components dissociate and allow passage of the plasmid expression vector in an episomal form. They can also fuse to the nuclear membrane releasing the vector directly into the nucleus. This method has been identified as the most suitable for producing recombinant cell lines by ensuring eGFP of pRP.ExBi-MTIIa-hPSA-eGFP and ZSGreen expression efficiency (**Figure 2**).

Three heavy metal salts have been reported as important inducers of protein expression mediated by MRE: cadmium, zinc, and copper. Cadmium presents high cellular toxicity. However, induction would be expected to occur at a low concentration (1 or 10 μM) and the cell death rate would be lower. Zinc can act as an analog of cadmium and compete for binding sites on the metallothionein or directly with the MTF-1 (Gunther et al., 2012). Copper also has the potential to bind to metallothioneins, but causes significant oxidative stress, which can also activate MTF-1 through accessory pathways such as phosphorylation (Furst et al., 1988; Suzuki et al., 2002; Thirumoorthy et al., 2011).

In our experiments, copper (CuSO_4_) was the agent that induced the highest expression level. Typically, zinc has a higher MTF-1 binding rate; therefore, low metal concentrations are sufficient to induce an increase in metallothionein expression. Contrary to this idea, herein, we report that a concentration as low as 100 μM of CuSO_4_ has an inducing capacity higher than that of zinc or cadmium. This suggests that oxidative stress acted synergistically to assist and activate MTF-1.

The effect of induction in the transfected cultures was evaluated over time. It was expected that expression would be stabilized and the cell death rate caused by the inductor would decrease. At this point, it was only possible to monitor hPSA-transfected cells over 72 h post-transfection. Our results confirmed that, after 24 h, the induced expression of eGFP was already higher than that in the non-induced control and increased until the trial timeout. Thus, there is a clear indication that eGFP expression is directly related to hPSA expression, which was being produced and secreted into the culture medium. This was confirmed by fluorescence microscopy.

BHK-21 cultures were the most promising, and although they were not as readily transfected as HEK293FT cells, these cells showed no morphological change and did not enter into the death phase like HeLa and HEK293FT cells. Furthermore, the hPSA production rate increased by approximately nine times compared with that in the other cultures.

The supernatant medium was collected and total hPSA was quantified using SDS-PAGE and ELISA. The assays confirmed that the protein was produced and that it possessed the corresponding native form. We found in the literature a method for inducing the MTIIa promoter (Jervis and Kilburn, 1996; Suzuki and Koizumi, 2000). In this study, the tested cell lines, which were of non-human origin, were more efficient in term of transfection, expression, and cell viability.

Cell viability is an important parameter to establish a stable, recombinant, and inducible culture. Disturbance in the environment caused by heavy metal addition decreased the rate of proliferation in almost all tested cell lines. The BHK-21 cell line, and to a lesser extent the CHO-K1 cell line, possessed greater resistance to the damage caused by heavy metal stimulation and showed low alterations of the rate of proliferation. This indicates that these cell types are more suitable for the production of a recombinant protein such as hPSA, because they ensure an initial expansion before induction and they can efficiently balance protein production and cell replication during induction. This is interesting from a biotechnological point of view because up-scale production of hPSA can be planned to obtain high yields of recombinant protein.

## Conclusion

We developed an inducible expression system based on the use of a plasmid expression vector containing a reduced region of the MTIIa promoter that included the hPSA gene modified for high efficiency processing and purification.

We determined the potential of three different metals (ZnCl_2_, CdCl_2_, and CuSO_4_) in inducing hPSA gene expression in four distinct cell lines (HEK293FT, HeLa, BHK-21, and CHO-K1 cells) transfected using three advantageous methods (MWCNT, Lipofectamine 2000, and X-tremeGENE HP Reagents). Our results indicate that BHK-21 cell line transfected with a non-cationic complex method showed lesser cell death in response to heavy metal treatment, and greater cell viability.

The hPSA was further compared with the native protein produced by LNCaP cells using ELISA and SDS-PAGE. The BHK-21 cell line showed the highest hPSA production rate. Furthermore, this cell line was more resistant to oxidative stress caused by 100 μM CuSO_4_. These results suggest that the proposed inducible expression system can effectively and efficiently produce complex mammalian recombinant proteins.

## Author Contributions

AS and RP participated in the design of the study, performed experiments, interpreted the results, and drafted the manuscript. RR designed the study, interpreted the results, and wrote the manuscript. All authors read and approved the final manuscript.

## Conflict of Interest Statement

The authors declare that the research was conducted in the absence of any commercial or financial relationships that could be construed as a potential conflict of interest.

## Abbreviations

AREs: antioxidant responsive elements
DMEM: Dulbecco’s Modified Eagle’s Medium
eGFP: enhanced green fluorescent protein
ELISA: enzyme-linked immunosorbent assay
FBS: fetal bovine serum
GREs: glucocorticoid responsive elements
hPSA: human prostate-specific antigen
IRES: internal ribosome entry site
MREs: metal-responsive elements
MTF-1: metal-binding transcription factor-1
MWCNT: Multi-walled carbon nanotubes
ROS: reactive oxygen species
SDS-PAGE: Sodium dodecyl sulfate polyacrylamide gel electrophoresis
SOC: super optimal broth
TEV: tobacco etch virus
